# Prediction of Intracranial Aneurysm Risk using Machine Learning

**DOI:** 10.1038/s41598-020-63906-8

**Published:** 2020-04-24

**Authors:** Jaehyuk Heo, Sang Jun Park, Si-Hyuck Kang, Chang Wan Oh, Jae Seung Bang, Tackeun Kim

**Affiliations:** 1 Department of Neurosurgery, Seoul National University Bundang Hospital, Seoul National University College of Medicine, Seongnam-si, Republic of Korea; 2 Department of Applied Statistics, The University of Suwon, Hwaseong-si, Republic of Korea; 3 Big Data Center, Department of Future Innovation Research, Seoul National University Bundang Hospital, Seongnam-si, Republic of Korea; 4 Department of Ophthalmology, Seoul National University Bundang Hospital, Seoul National University College of Medicine, Seongnam-si, Republic of Korea; 5 Division of Cardiology, Department of Internal Medicine, Seoul National University Bundang Hospital, Seoul National University College of Medicine, Seongnam-si, Republic of Korea

**Keywords:** Epidemiology, Risk factors

## Abstract

An efficient method for identifying subjects at high risk of an intracranial aneurysm (IA) is warranted to provide adequate radiological screening guidelines and effectively allocate medical resources. We developed a model for pre-diagnosis IA prediction using a national claims database and health examination records. Data from the National Health Screening Program in Korea were utilized as input for several machine learning algorithms: logistic regression (LR), random forest (RF), scalable tree boosting system (XGB), and deep neural networks (DNN). Algorithm performance was evaluated through the area under the receiver operating characteristic curve (AUROC) using different test data from that employed for model training. Five risk groups were classified in ascending order of risk using model prediction probabilities. Incidence rate ratios between the lowest- and highest-risk groups were then compared. The XGB model produced the best IA risk prediction (AUROC of 0.765) and predicted the lowest IA incidence (3.20) in the lowest-risk group, whereas the RF model predicted the highest IA incidence (161.34) in the highest-risk group. The incidence rate ratios between the lowest- and highest-risk groups were 49.85, 35.85, 34.90, and 30.26 for the XGB, LR, DNN, and RF models, respectively. The developed prediction model can aid future IA screening strategies.

## Introduction

Intracranial aneurysm (IA) is a cerebrovascular disease that predominantly occurs in the cerebral artery and is characterized by pathologic dilatation of blood vessels. A rupture of IA induces a subarachnoid hemorrhage (SAH), a type of hemorrhagic stroke that frequently leads to death or severe disability. According to a recent report, the incidence of SAH is largely stable, whereas the incidence of unruptured IA (UIA) has markedly increased owing to increased healthcare screening^1–4^.

Although a large proportion of IAs are diagnosed as UIAs during medical check-ups, the costs and risks associated with cerebrovascular examinations make screening the entire population unfeasible^5^. Thus, stratifying the risk of developing IA is necessary to select only the most relevant subjects for screening. Current guidelines for UIA screening in the United States and Korea contain only two categories: 1) patients with at least 2 family members with UIA or SAH, and 2) patients with a history of autosomal dominant polycystic kidney disease (ADPKD), coarctation of the aorta, or microcephalic osteodysplastic primordial dwarfism^6^. However, considering the prevalence of UIA^7,8^ and the proportion of the population with familial SAH history and ADPKD^9^, the coverage of current guidelines is likely to be very limited.

The majority of studies on IA are focused on the rupture risk of UIA, and only a few studies have focused on the risk of IA development^2,10,11^. Thus, risk prediction of UIA development using non-invasive healthcare screening data can supplement the limitations of current guidelines and contribute to the improvement of healthcare policies. Owing to the relatively low incidence of IA, a large dataset such as a national database is required to predict IA risk. Thus, the National Health Insurance Service-National Sample Cohort (NHIS-NSC), provided by the National Health Insurance Service (NHIS) in Korea, which consists of medical billing and claims data, as well as general health examination results, can be a suitable data source for predicting the risk of disease^2,12–16^.

Recently, many machine learning algorithms have been developed and applied to disease risk prediction and have shown improved performance when combined with big data^17–19^. Similarly, verifying predictive power beyond conventional statistical methods and overcoming class imbalance would significantly supplement the limitations of current screening guidelines for measuring individual risk of UIA before rupture. In this study, several machine learning algorithms were evaluated utilizing the results of general health examinations including anthropometric data, blood pressure measurements, and laboratory data derived from NHIS-NSC for risk prediction of IA development.

## Methods

### Data extraction

The National Health Information Database (NHID) is a public database organized by NHIS that covers approximately 50 million people or 97% of the entire population of Korea. It includes information on healthcare utilization, sociodemographic status, and mortality data. Moreover, it contains the results of general health examinations provided by NHIS at least once every two years for all subscribers. The NHIS-NSC represents the entire population and was created by randomly selecting 2% of the population by stratification as a sample cohort. The NHIS-NSC comprises four databases and includes participant insurance eligibility, medical treatments, general health examinations conducted by NHIS, and lifestyle and behavioral information obtained from questionnaires. A detailed data profile was published by the Big Data Steering Department of the NHIS^20^. The NHIS review board approved all data requests for research purposes (NHIS-2019-2-083). Because this public database is fully anonymized, institutional approval was waived by the institutional review board (X-2019/522-903).

We extracted data of subjects who underwent general health examinations from 2009 to 2013 from the NHIS-NSC. This time period was selected because major changes were made to health examination screening and questionnaires in 2009 after a system restructure. For subjects who underwent multiple general health examinations, only their earliest record was considered. General health examinations consisted of a medical interview, postural examination, blood test, and urine test. All test results were linked with an anonymized identification key for healthcare utilization, including a diagnosis code, sociodemographic status, and mortality data. To estimate IA incidence, the index date was set as the first day of the general health examination year. The end of the observation period was set to December 31, 2013.

**Figure 1:**
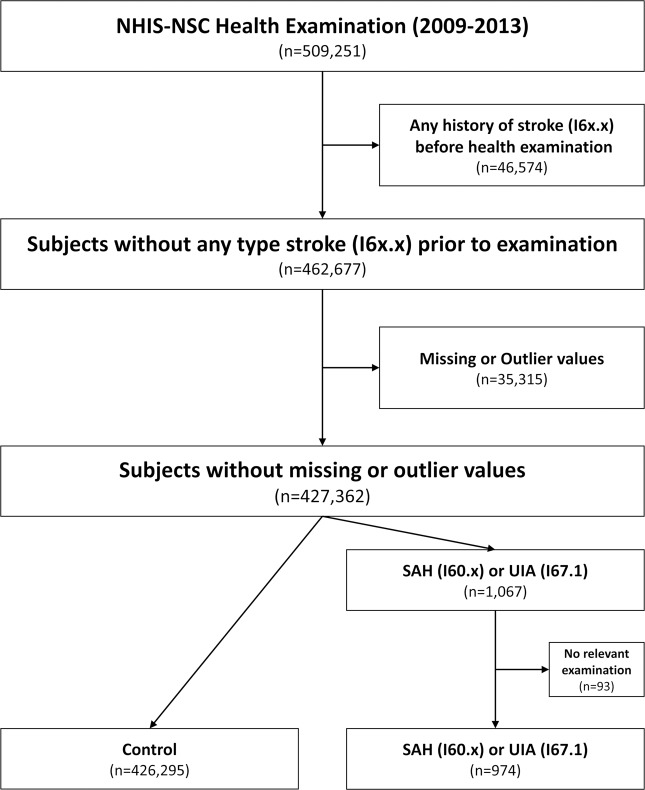
Flowchart of the data processing strategy. NHIS-NSC = National Health Insurance Service-National Sample Cohort; SAH = subarachnoid hemorrhage; UIA = unruptured intracranial aneurysm.

Among the 509,251 subjects screened, 46,574 subjects diagnosed with a stroke prior to the examination, including SAH and UIA, were excluded. Any subjects with outliers more than four times the standard deviation of each continuous set of data or with any missing values were also excluded. Among the eligible 427,362 subjects, 1,067 were identified using the diagnostic codes UIA (I671) or SAH (I60x), and the remaining 426,295 subjects were allocated to the control group. Finally, 974 subjects were allocated to the IA group after excluding 93 patients who had not undergone computed tomography (CT), magnetic resonance (MR), or cerebral angiography correlated with the diagnostic codes (Fig. 1).

Twenty-one variables were included in the health examination data: age, sex, BMI, waist circumference, SBP, DBP, FBS, total cholesterol, high-density lipoprotein (HDL), low-density lipoprotein (LDL), triglyceride (TG), hemoglobin, creatinine, gamma-glutamyl transferase (GGT), aspartate aminotransferase (AST), alanine aminotransferase (ALT), smoking status (never, ex-, or current-smoker), and familial histories of stroke, hypertension, heart disease, and diabetes.

For model training and evaluation, we separated all subjects into training (70%) and test (30%) datasets through random allocation. 299,088 subjects were allocated to the training dataset, which included 682 (0.2%) IA cases, and 128,181 were allocated to the test dataset, which included 292 (0.2%) IA cases.

### Prediction models and evaluation

Logistic regression (LR), random forest (RF)^21,22^, scalable tree boosting system (XGB)^23,24^, and deep neural network (DNN)^25^ were used as the machine learning algorithms for classification. All training processes were performed using ten-fold cross-validation^26^. Model performance was evaluated with the separate test dataset using the area under the receiver operating characteristic curve (AUROC), which consisted of plots of trade-offs between sensitivity and 1-specificity across a series of cut-off points. Each parameter related to the training process was determined by grid searching to achieve the highest AUROC. The parameters for RF were explored for 100, 300, 500, 700, and 1,000 trees with a maximum depth between 3 and 5. The optimal parameters for XGB were determined after experimenting with a total of 108 combinations with learning rates of 0.1, 0.5, 1.0; a maximum depth between 4 and 6; a minimum child weight between 3 and 6; and subsample rates of 50%, 80%, and 100%. For DNN, we tested a total of 162 combinations for DNN training using 1, 3, and 5 hidden layers; each layer taken into account with 128, 512 and 1,280 trainable nodes; learning rates of 0.0001, 0.001, and 0.01; and batch sizes of 1,024, 2,048, and 4,096. Lastly, we tested two loss functions: binary cross entropy and focal loss to compensate for case number imbalance^27^.

### Statistical methods

To verify that the training and test data were distributed equally, we conducted Student's t-tests for continuous variables and Pearson's chi-squared tests for categorical variables. The method of DeLong *et al*. was used to test statistically significant differences among the AUROC values of each model^28^.

Based on the prediction probability of each model, the risk score was scaled from zero (lowest-risk) to one (highest-risk) for each model. Subjects of the test dataset were divided into five groups in ascending order of their risk score. Quintiles were divided into lowest-risk, lower-risk, mid-risk, higher-risk, and highest-risk groups. Incidence of IA was calculated as $\frac{Number\,of\,cases}{Total\,observation\,size\,(person\,\times \,year)}\times 100,000$ and presented per 100,000 person-years. Additionally, survival analysis was performed using a log-rank trend test with pair-wise Bonferroni correction among the groups. Correlations among the variables according to prediction score were identified using Pearson correlation tests. Generally, a p-value under 0.05 was considered statistically significant.

## Results

As shown in Table 1, there were no statistical differences between the training and test datasets.

**Table 1:** Factor distribution differences between training and test datasets.

Factors	Training set (n = 299,088)	Test set (n = 128,181)	p-value
Age	46.05 ± 13.92	46.04 ± 13.94	0.93
Sex (Female)	152,746 (51.1)	65,677 (51.2)	0.32
BMI	23.55 ± 3.28	23.55 ± 3.29	0.87
Waist circumference	79.41 ± 9.29	79.41 ± 9.31	0.98
Hypertension	39,341 (13.2)	16,248 (13.0)	0.23
Systolic BP (mmHg)	121.17 ± 14.66	121.18 ± 14.68	0.92
Diastolic BP (mmHg)	75.54 ± 9.91	75.56 ± 9.93	0.58
DM	13,686 (4.6)	5,754 (4.5)	0.21
Glucose (mg/dl)	95.1 ± 16.19	75.56 ± 9.93	0.61
Total cholesterol (mg/dl)	193.64 ± 35.83	193.67 ± 35.88	0.81
LDL cholesterol (mg/dl)	55.81 ± 13.79	55.85 ± 13.81	0.33
HDL cholesterol (mg/dl)	113.21 ± 32.79	113.24 ± 32.85	0.79
Triglyceride (mg/dl)	122.4 ± 72.9	122.28 ± 72.61	0.62
Hemoglobin (g/dl)	13.87 ± 1.61	13.86 ± 1.61	0.52
Creatinine (mg/dl)	0.89 ± 0.22	0.89 ± 0.22	0.67
AST (IU/L)	23.75 ± 8.74	23.74 ± 8.72	0.75
ALT (IU/L)	22.96 ± 14.09	22.97 ± 14.11	0.73
GGT (IU/L)	31.36 ± 28.9	31.31 ± 28.85	0.59
Smoking			
Never	187,333 (62.6)	80,371 (62.7)	0.68
Ex	37,987 (12.7)	16,248 (12.7)	0.83
Current	73,768 (24.7)	31,562 (24.6)	0.78
Familial history of stroke	16,590 (5.5)	7,098 (5.5)	0.91
Familial history of heart disease	10,320 (3.5)	4,351 (3.4)	0.36
Familial history of hypertension	34,574 (1.6)	14,982 (1.7)	0.23
Familial history of diabetes	27,424 (9.2)	11,805 (9.2)	0.68

Continuous variables are presented as mean ± standard deviation. Categorical variables are represented as numbers (percentages). BMI = body mass index; BP = blood pressure; DM = diabetes mellitus; AST = aspartate aminotransferase; ALT = alanine transaminase; GGT = gamma-glutamyl transferase.

**Figure 2:**
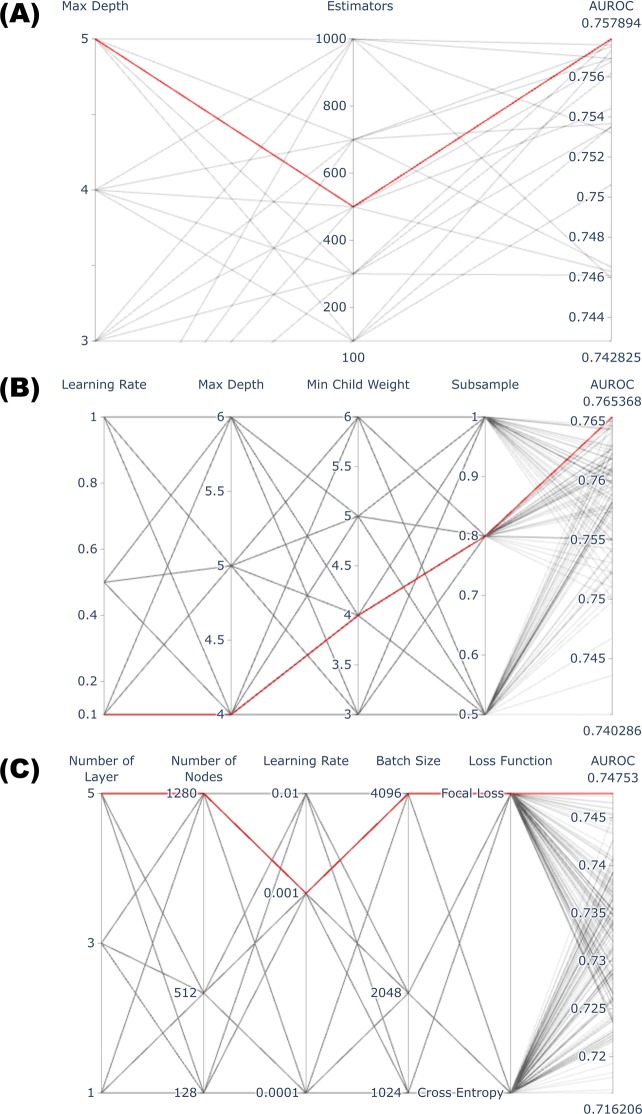
Summary of grid search process for optimizing parameters. Each line shows the combination of parameters used in the grid search. The thick red line indicates the optimal combination of parameters achieving the highest AUROC (area under receiver operating characteristic curve).

The highest AUROC (0.762) was obtained using the LR model with L2 regularization. An AUROC of 0.757 (Fig. 2A) was achieved using the RF model with maximum depth and number of trees set to 5 and 500, respectively. An AUROC of 0.765 was obtained by performing a grid search for XGB training, applying a learning rate of 0.1, maximum depth of 4, minimum child weight of 4, and 80% subsampling (Fig. 2B). An AUROC of 0.748 was achieved among the test dataset with the DNN model; seven layers, including five hidden layers, gave the optimal structure with a learning rate of 0.001 and a batch size of 4,096 (Fig. 2C). The numbers of trainable nodes in each hidden layer were 128, 256, 512, 256, and 128, in order, producing 332,289 trainable parameters throughout the entire network.

Table 2 shows the performance indicators for each model. Although within the margin of error for the LR model (p = 0.485), the XGB model exhibited superior IA risk prediction performance compared to the RF (p = 0.049) and DNN (p = 0.010) models. For risk prediction using the XGB model, age was the most important feature (relative importance = 1.00), followed by BMI (0.36 ± 0.08), triglyceride (0.34 ± 0.08), hypertension (0.32 ± 0.14), and total cholesterol (0.31 ± 0.08). The five most important features of LR were age (relative importance = 1.00), familial history of stroke (0.73 ± 0.06), sex (0.66 ± 0.04), familial history of hypertension (0.41 ± 0.07), and familial history of heart disease (0.32 ± 0.09), differed from XGB model. Moreover, age (relative importance = 1.00), systolic blood pressure (SBP; 0.12 ± 0.01), hemoglobin (0.11 ± 0.01), triglyceride (0.10 ± 0.01), and BMI (0.09 ± 0.01) were the five most important features for prediction through the RF model. The relative importance of each feature of the DNN model was calculated using guided backpropagation^29^. Similar to the other models, age showed the highest importance (relative importance = 1.00) followed by total cholesterol (0.59 ± 0.12), familial history of diabetes (0.59 ± 0.13), familial history of stroke (0.57 ± 0.13), and diabetes mellitus (0.57 ± 0.13).

**Table 2:** Model performance indicators.

Model	AUROC	95% confidence interval	Sensitivity	Specificity	p-values		
					vs. LR	vs. RF	vs. DNN
XGB	0.765	0.742–0.788	0.805	0.613	0.485	0.049	0.010
LR	0.762	0.739–0.784	0.788	0.621	—	0.487	0.021
RF	0.757	0.733–0.779	0.815	0.591		—	0.197
DNN	0.748	0.724–0.770	0.853	0.571			—

AUROC = area under the receiver operating characteristic curve; LR = logistic regression; RF = random forest; DNN = deep neural networks; XGB = scalable tree boosting system.

**Figure 3:**
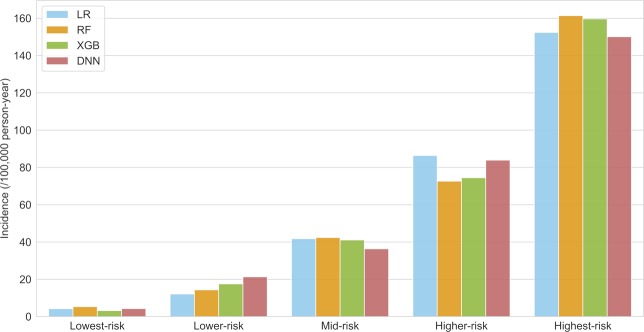
IA incidence according to each risk group. IA incidence per 100,000 person-year by quintile risk group. IA = intracranial aneurysm; LR = logistic regression; RF = random forest; XGB = scalable tree boosting system; DNN = deep neural networks.

All subjects in the test dataset were grouped into quintile risk groups according to the probability scores derived from each model. Figure 3 summarizes the incidence of IA according to risk group for each model. Within the lowest-risk group, the XGB model showed the lowest IA incidence (3.20 [95% CI, 0.83–10.20]), followed by the LR (4.25 [1.36–11.68]) and DNN (4.30 [1.38–11.83]) models. Conversely, the RF model showed the highest IA incidence within the highest-risk group (161.34 [137.68–187.10]), followed by the XGB (159.59 [136.05–185.52]) and LR (152.31 [129.36–179.24]) models. The incidence rate ratios between the lowest- and highest-risk groups were 49.85 (15.90–156.22), 35.85 (13.28–96.77), 33.38 (12.36–90.17), and 30.26 (12.42–73.69) for the XGB, LR, DNN, and RF models, respectively.

**Figure 4:**
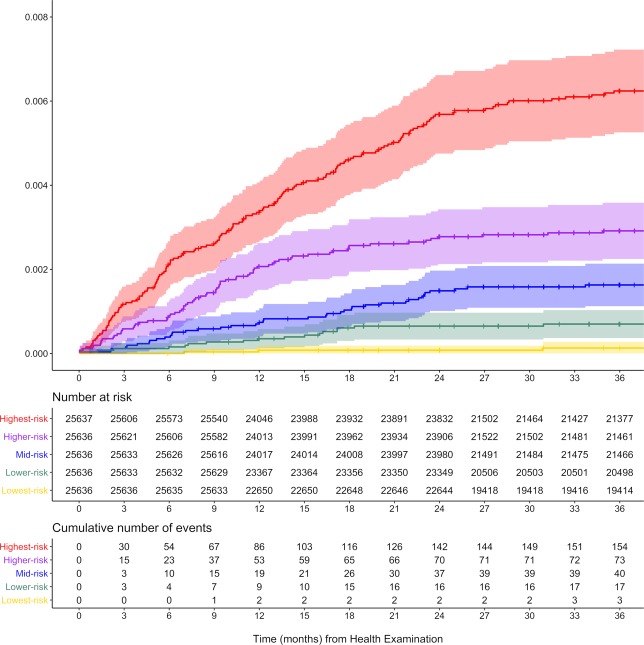
Survival curves for IA incidence by risk group predicted by scalable tree boosting systems (XGB).

Considering the AUROC values and rate-ratios between the lowest- and highest-risk groups, we identified the XGB model as the best classifier for further analysis. Survival analysis of the risk groups derived from the XGB model demonstrated statistical differences among the groups (Fig. 4). The three-year cumulative incidence of IA was 0.62% (95% CI, 0.53–0.72), 0.29% (0.22–0.36), 0.16% (0.11–0.21), 0.07% (0.04–0.10), and 0.01% (0.00–0.03) for the highest-, higher-, mid-, lower-, and lowest-risk groups, respectively (p < 0.01). Moreover, Bonferroni adjustments for each group were statistically significant (p < 0.01) for each pair-wise comparison.

**Figure 5:**
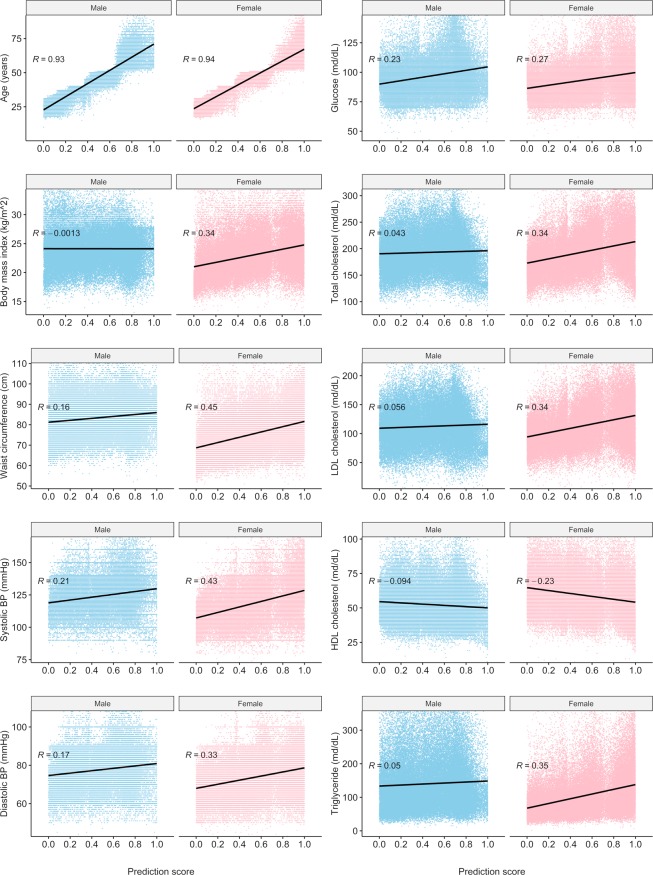
Distribution of variables according to IA prediction score by sex. R = Pearson correlation coefficient.

Correlation tests between prediction scores and continuous variables are summarized in Fig. 5. For both sexes, age showed a strong positive correlation with prediction score. Conversely, the relationship between BMI and prediction score showed a dependence on sex. This means that the correlation coefficient (R) was 0.34 for females but almost zero for males. Females exhibited stronger positive correlations than males for waist circumference, SBP, and diastolic blood pressure (DBP). Both males and females showed a similar positive correlation with fasting blood glucose (FBS; R = 0.23 for males, 0.27 for females). Males showed little correlation (−0.1 < R < 0.1) with lipid panel results, whereas females demonstrated positive correlations with total cholesterol, LDL, and TG, while having a negative correlation with HDL.

According to the XGB model, the feature importance of variables, including hemoglobin, creatinine, liver function tests, and family histories, was very low. Hypertension was ranked as an important feature (relative importance = 0.32 ± 0.14), whereas diabetes mellitus was ranked as unimportant (0.08 ± 0.16). Smoking status was also determined to be an unimportant variable for IA risk assessment.

## Discussion

Several diagnostic imaging modalities have been used for detecting IA. Although cerebral angiography is considered the optimal diagnostic technique, the rate of complication is not negligible^30^. CT angiography also requires a contrast agent, which can cause contrast-induced nephropathy^31^. In addition, the patient is exposed to radiation in both modalities^32^. Although MR angiography is relatively safe from these risks, the associated medical costs are very high^33^. Thus, screening for IA should be recommended for selected high-risk subjects. Current screening guideline coverage is overly selective for subjects with strong familial history or several genetic diseases, despite the majority of IA patients not having such risk factors^2,6,9^. Considering the growing number of health examinations comprising relatively safe laboratory tests and measurements, risk assessment using these data would be valuable to enhance decision making.

In this study, we evaluated several prediction models to stratify the risk of IA development using health examination data. Data related to low-incidence diseases are unevenly distributed, which inevitably induces problems for machine learning. If we select classification accuracy as an indicator of performance, the model that predicts no occurrence of disease among all cases can easily achieve an accuracy in excess of 99%^34^. Thus, we adopted the AUROC ranking method for performance evaluation.

**Figure 6:**
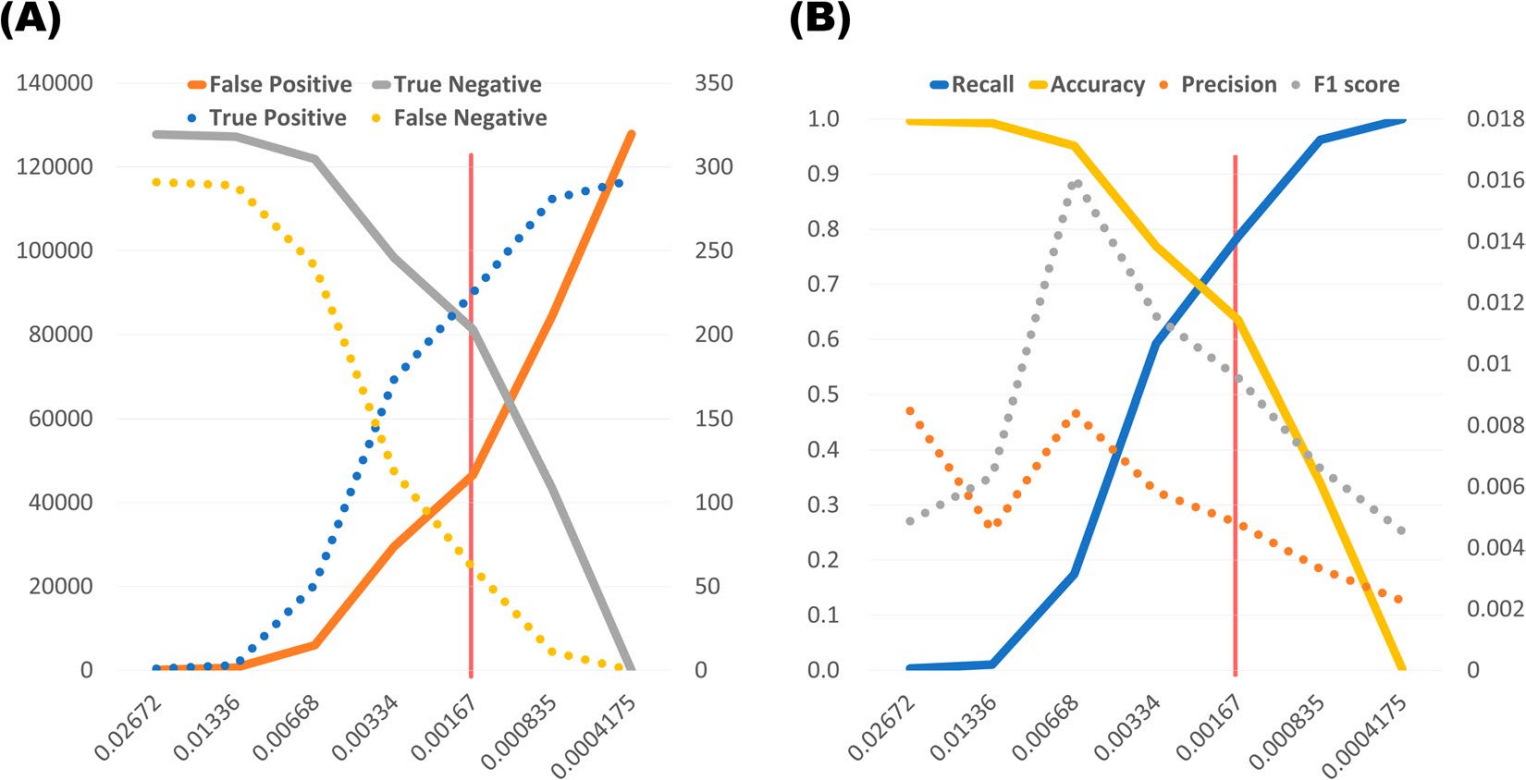
Trade-off line graph according to various cut-off probabilities calculated by XGB model. The x-axis indicates probability values calculated from the model. The scale of the solid line is shown on the left axis, and the scale of the dotted line is shown on the right axis. The red vertical line indicates the optimal cut-off value maximizing sensitivity + specificity. (A) Trends of the number of predictions. The y-axis represents the number of subjects. (B) Trends of performance indicators. The y-axis represents the performance scores.

Among the four models trained in this study, the highest AUROC value was achieved by the XGB algorithm (0.765) using separate blind test data. The risk score was scaled from zero to one. Originally, all models were designed as binary classifiers to predict patient groups from the general population. However, owing to extreme class imbalance, a 0.5 probability cut-off produced 0.998 accuracy with 0 cases of positive prediction. The optimal probability cut-off for maximizing sensitivity + specificity was 0.00167. However, at this cut-off point, the numbers of true and false positive predictions were 225 and 46,644, respectively, yielding a precision score of 0.0048 (Fig. 6). Therefore, comparative analysis was conducted by setting cut-off values dividing quintiles by probability score for a more practical risk prediction. The XGB prediction model showed an incidence rate ratio of 49.85 (95% CI, 15.90–156.22) between the highest- and lowest-risk groups. In other words, the highest-risk group assessed by the XGB model had a 50 times greater risk of developing IA than the lowest-risk group. Considering the number of cases, 53.8% (157 of 292) of IA patients belonged to the highest-risk group (20.0%, 25,637 of 128,181).

Compared to the well-known statistical method, interpreting the results of XGB is more complex. Each variable was evaluated based on its feature importance, i.e., the relative contribution to the model was calculated using the contribution of each tree in the model. As this metric indicates the relative importance of each variable for generating a prediction, we assigned a value of 1.0 to the variable with the highest importance (age) and relative values for the other variables. Age was consistently the most important feature. The mean age of the highest group was 64.07 ± 7.86 years and ranged from 35–96 years. Although smoking has previously been considered one of most important modifiable risk factors for SAH or rupture from known UIA^6^, our model underestimated the importance of this factor. In fact, the NHIS-NSC overestimated the proportion of female non-smokers compared to the general population^20^. Thus, previous investigations of IA risk factors using NHIS-NSC data also revealed that lifestyle factors (smoking, drinking, and exercise) were eliminated from multivariate Cox regression model analyses^2^. Owing to the low incidence of IA, which required a large dataset for model training, NHIS-NSC general health examination data should be an effective data source, despite its limitations. For these reasons, only two previous studies have reported potential UIA risk factors before diagnosis in an unselected population^2,35^.

Most UIAs are likely to remain undetected owing to the high cost and invasiveness of radiological assessments, particularly considering the low detection rate of IA by MR angiography (approximately 5%)^3,7,8^. Therefore, an efficient method of identifying high-risk subjects is required to provide adequate screening services and effectively allocate limited medical resources. In this study, we used a large dataset derived from a universal insurer covering more than 97% of the population in Korea, of which its representativeness has been previously discussed^20^. Although single or multi-institutional databases based on medical records tend to be vulnerable to selection bias, results from general health examinations provided by NHIS covered all subscribers without selection.

However, because of the nature of the dataset employed, caution should be exercised in its global application. The incidence of SAH is known to be higher in the Western Pacific Region (including Korea) than in other regions. Moreover, the incidence of UIA in Korea is also markedly higher than that in other countries^2,16,36,37^. Considering potential reasons for discrepancies, the global applicability of this model requires further validation^38^. Another limitation of this study is related to the accuracy of IA diagnosis; the dataset used in this study did not contain relevant medical images for determining specific diagnoses and aneurysm characteristics. To overcome this limitation, we excluded subjects diagnosed with IA who did not undergo CT, MR, or cerebral angiography examinations within 14 days of diagnosis in an attempt to extract only definite IA cases. Moreover, IA diagnoses were strictly reviewed by the Health Insurance Review and Assessment Service in Korea. Nevertheless, it should be noted that the accuracy of the NHID for the diagnosis of severe illnesses, such as ischemic strokes and myocardial infarction, is below 85%^39,40^.

The results of this study may provide information on the relative risk of IA development for those conducting health examinations. According to risk stratification, adequate screening tests can be recommended even if they are not covered by current guidelines. Despite some limitations, the proposed XGB model exhibited considerable scope for estimating IA risk. However, model enhancement and further validation using prediction data feedback should be considered to produce a more robust prediction model for updating current screening guidelines. Additionally, cost-effectiveness analysis is warranted to provide dependable consult for IA risk using healthcare examination results.

## Data Availability

The NHIS-NSC database is available for research purposes approved by the data provision review committee of the National Health Insurance Service.
